# Delayed Tension Hemothorax With Nondisplaced Rib Fractures After Blunt Thoracic Trauma

**DOI:** 10.7759/cureus.38835

**Published:** 2023-05-10

**Authors:** Nana Hamamoto, Shota Kikuta, Ryo Takahashi, Satoshi Ishihara

**Affiliations:** 1 Emergency and Critical Care Medicine, Hyogo Emergency Medical Center, Kobe, JPN

**Keywords:** extravasation, motorcycle accident, intercostal artery, rib fractures, emergency thoracic drainage, chest drainage, obstructive shock, delayed hemothorax, tension hemothorax, blunt thoracic trauma

## Abstract

Blunt thoracic trauma often causes rib fractures, hemothorax, and pneumothorax. Although there is no established definition regarding the duration and management of delayed hemothorax, it commonly occurs in a few days and exhibits at least one displaced rib fracture. Moreover, delayed hemothorax rarely develops tension hemothorax. A 58-year-old male who had a motorcycle accident received conservative treatment from his orthopedic doctor. He felt a sudden severe chest pain 19 days after the accident. Contrast-enhanced computed tomography (CT) of the chest revealed multiple left-sided rib fractures without displacement, left pleural effusion, and extravasation near the intercostal space of the seventh rib fracture. After transfer to our hospital and a plain CT scan, which showed a more mediastinal shift toward the right, his condition deteriorated with cardiorespiratory embarrassment, such as restlessness, hypotension, and neck vein distention. We diagnosed him with obstructive shock due to tension hemothorax. Immediate chest drainage ameliorated restlessness and elevated blood pressure. Here, we report an extremely rare and atypical case of delayed tension hemothorax after blunt thoracic trauma without displaced rib fractures.

## Introduction

Blunt thoracic trauma often causes rib fractures. Hemothorax and pneumothorax are also frequent findings. Delayed hemothorax after blunt thoracic trauma is rare and commonly occurs in a few days, but there is no established definition regarding the duration of delayed hemothorax. Sharma et al. reported that hemothorax sometimes occurred at a later time in seven (4.2%) of 167 patients with blunt thoracic trauma in their eight-year retrospective analysis; however, most of them occurred within 22-95 hours [1]. Here, we present the case of a 58-year-old male as the first case of delayed tension hemothorax that developed more than two weeks after blunt thoracic trauma without displaced rib fractures.

## Case presentation

**Figure 1:**
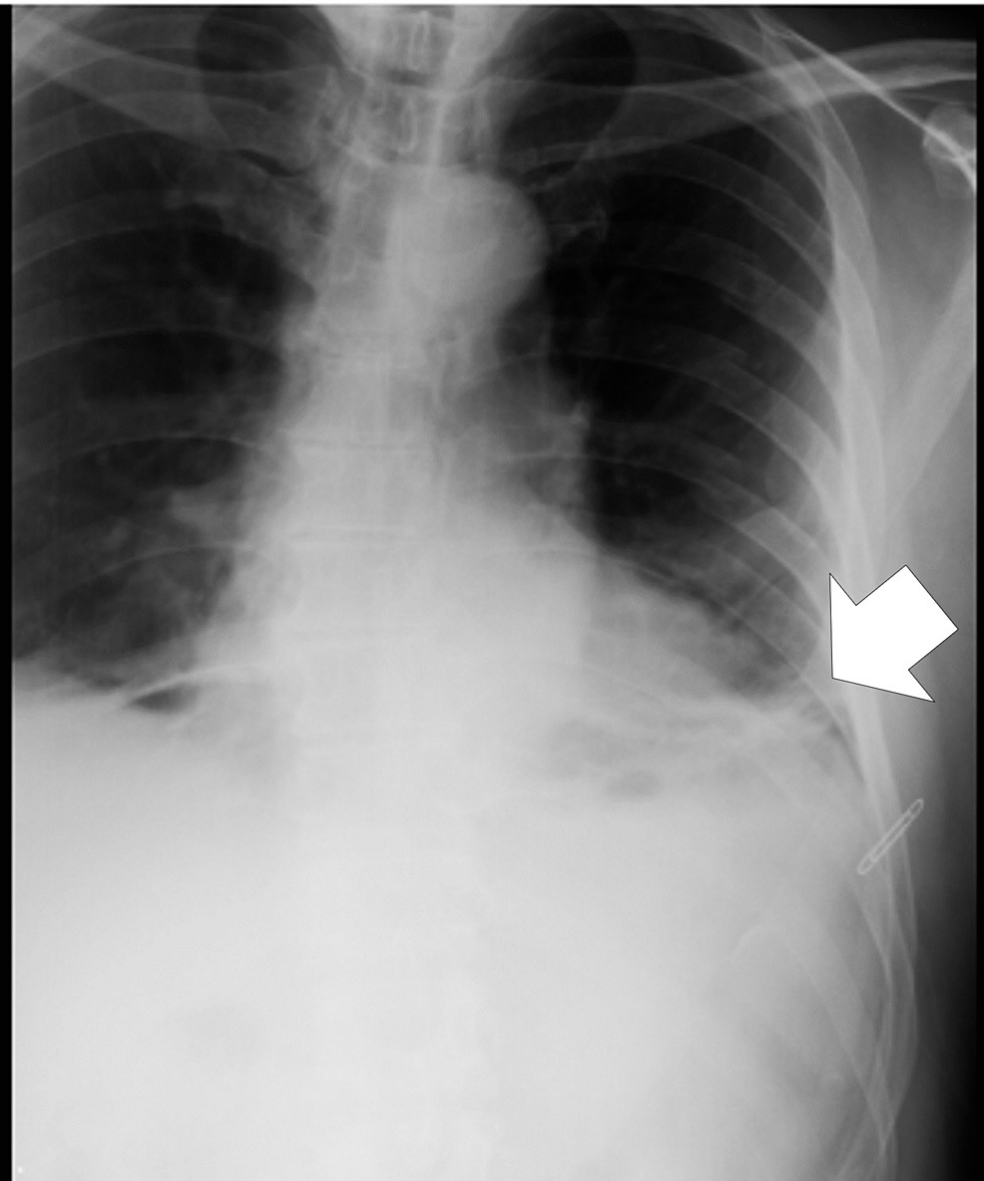
Anteroposterior chest X-ray image showing the persistence of trivial left pleural effusion performed at a family doctor (four days after the accident).

**Figure 2:**
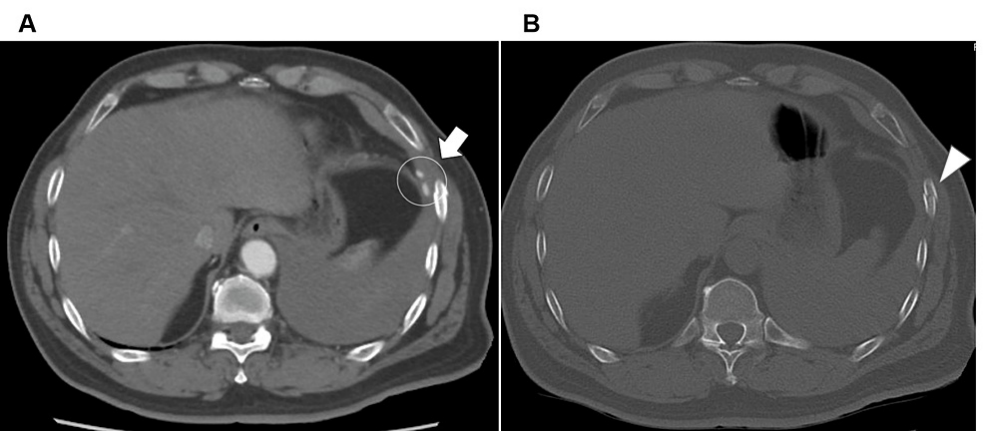
Contrast-enhanced CT scan of the chest performed at the referring hospital. A: Axial scan of arterial phase showing a left pleural effusion and extravasation near the intercostal space of the seventh rib fracture. B: Axial scan of the bone window showing the seventh rib fracture.

A 58-year-old man, who had no past medical or family history of coagulation disturbance and did not take any anti-coagulation treatment, saw a family doctor (orthopedics) four days after a motorcycle accident because of persistent left-sided rib pain. His chest X-ray image (anteroposterior view of the ribs) for checking rib fractures showed the persistence of trivial left pleural effusion (Figure 1). For this, he regularly visited the clinic to receive conservative medical treatment, but a follow-up chest X-ray was not conducted during the visit. The pain improved, but exertional dyspnea worsened slightly. On the 19th day post-trauma, he felt a sudden strong left-sided chest pain while driving his motorcycle and presented to the referring hospital. Computed tomography (CT) revealed multiple left-sided rib fractures (fifth to eighth ribs) without dislocation, left pleural effusion, and active extravasation near the intercostal space of the seventh rib fracture (Figures 2A, 2B). Therefore, he was transferred to our hospital.

**Figure 3:**
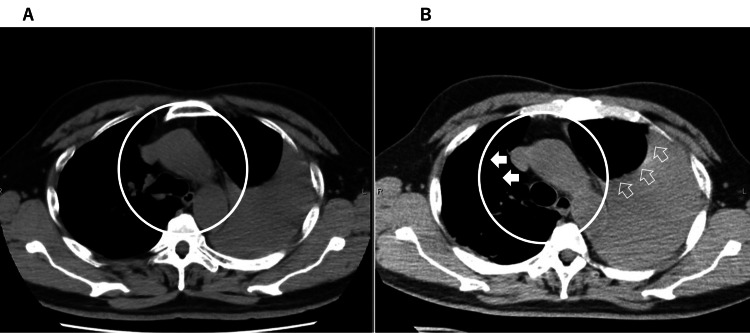
CT scan showing a slightly bigger hemothorax with a mediastinal shift performed at our hospital (two hours after obtaining Figure 2). A: CT scan performed at the referring hospital (taken at almost the same time as Figure 1). B: CT scan performed at our hospital.

His vital signs were as follows: Glasgow Coma Scale (GCS) score of 15/15; blood pressure of 107/70 mmHg; heart rate of 106 beats per minute; respiratory rate of 26 breaths per minute; and oxygen saturation of 95% with 7 L/minute oxygen via oxygen mask. CT performed in our hospital showed a slightly bigger hemothorax with mediastinal shift (Figures 3A, 3B). Left chest drainage was planned.

When chest drainage was started, the patient’s condition deteriorated with hypotension and cardiorespiratory embarrassment, such as restlessness, hypotension decreasing in pulse pressure, and distension of the external jugular vein. Therefore, we considered his condition as an obstructive shock.

We inserted a chest tube, which immediately yielded approximately 1,000 mL of dark red blood. As a result, his blood pressure elevated, and neck vein distension and tachypnea improved. Thereafter, emergency interventional radiology revealed no extravasation in the thoracic cavity, but a seventh intercostal arterial spasm was detected. Therefore, we determined the vessel as the responsible bleeding site and performed transcatheter arterial embolization. Subsequently, he was admitted to the intensive care unit (ICU).

During his stay in the ICU for observation, 1,600 mL of bloody fluid was evacuated in total, and the chest tube was removed on day three. He was discharged home in stable condition on day seven without adverse events.

## Discussion

To our knowledge, this is the first case of tension hemothorax in an adult without displaced rib fractures that developed more than two weeks after blunt thoracic trauma. The acute-phase hemorrhage for some reason caused the delayed-onset tension hemothorax on chronic hemothorax. Hence, blunt thoracic trauma might cause delayed tension hemothorax even if the injury occurred over two weeks earlier and rib fractures were not displaced.

According to Advanced Trauma Life Support guidelines, tension pneumothorax is a clinical diagnosis reflecting air under pressure in the affected pleural space and is characterized by some or all of the following signs and symptoms: chest pain, air, hunger, tachypnea, respiratory distress, tachycardia, hypotension, tracheal deviation away from the side of the injury, unilateral absence of breath sounds, elevated hemothorax without respiratory movement, neck vein distention, and cyanosis. Although there is no definition of tension hemothorax, findings such as chest pain, tachypnea, hypotension, and neck vein distention may indicate tamponade or tension physiology [2,3]. Although the mediastinal shift appears moderate in Figure 3B, it is possible that tension hemothorax was further advanced during preparation for thoracic drainage.

Tension hemothorax is typically caused by thoracic penetrating or blunt trauma or ruptured thoracic aortic aneurysm [4,5]. Fourdrain et al. reported delayed-onset tension hemothorax following blunt trauma and the rupture of a previously undiagnosed aberrant right subclavian artery [6]. Muronoi et al. reported a rare case of delayed massive hemothorax due to a diaphragm injury 17 hours after blunt trauma [7]. Piastra et al. reported delayed tension hemothorax after chest trauma in children [8]. Interventional radiology did not show pseudoaneurysm or extravasation of contrast material, but there was an angiospasm of the seventh intercostal artery near the rib fractures. Therefore, we indirectly determined that the intercostal artery was the source of bleeding.

Delayed hemothorax is thought to be caused by a secondary indirect injury rather than a direct external force [9]. There is a report of delayed hemothorax that occurred after a severe coughing spell or intermittent positive pressure breathing [10]. Geoffrey et al. reported that the posterior location and displacement of at least one rib fracture in the initial CT were independent risk factors for predicting the occurrence of delayed hemothorax [11], but our patient’s rib fractures were not displaced and located anteriorly. Here, the cause of the delayed hemothorax remains unknown, but we believe that it might be due to the vibration of the motorcycle, respiratory movement, or the fracture site itself. Thus, this case is very rare in two ways, namely, the tension hemothorax was mainly derived from the intercostal artery, and there was no dislocation of the nearby rib fracture, which led to delayed hemothorax.

Thoracic trauma may include cases of rib fractures, pneumothorax, or hemothorax [1]. However, there are only a few cases of delayed hemothorax. Misthos et al. detected pleural blood collections in 52 (7.4%) of 902 patients up to 14 days after injury despite normal clinical and radiologic findings during the first 36 hours post-trauma [12]. The definition of delayed hemothorax regarding onset time or pathology is not established yet. Ritter et al. defined it as hemothorax that occurred more than two hours after the injury if there were no findings of hemothorax at the time of initial examination [13]. Shorr et al. defined it as hemothorax that occurred 24 hours after injury [1]. Sharma et al. did not define it clearly but mentioned that delayed hemothorax had been reported on repeat chest radiograph studies within three to 24 hours of hospital observation of stab wounds [14]. They showed that delayed hemothorax occurred in only seven (4.2%) of 167 patients. Among the seven patients, six had delayed hemothorax within 22 to 95 hours, and all cases of delayed hemothorax occurred within 16 days [14]. Therefore, our case of hemothorax that occurred 19 days after injury was considered extremely rare. We assumed that in our case acute-phase hemorrhage on chronic mild-to-moderate hemothorax developed obstructive shock. The anteroposterior chest X-ray image on day one showed the persistence of trivial left pleural effusion, and the patient felt worsening exertional dyspnea several days before the sudden chest pain; thus, this shortness of breath might be affected by small amounts of pleural effusion. Because the drainage showed dark red blood, this hemothorax was considered to include not only arterial but also venous blood or some exudative pleural effusion. Similarly, based on the fact that the patient was going about his daily life, it was unlikely that arterial bleeding had persisted immediately after the injury.

## Conclusions

This is the first case report of delayed tension hemothorax without displaced rib fractures after blunt thoracic trauma in an adult. In this case, additional acute-phase hemorrhage on chronic hemothorax caused the delayed-onset tension hemothorax. Therefore, blunt thoracic trauma with nondisplaced rib fractures should be carefully evaluated because it might cause tension hemothorax even more than two weeks after the event.
